# Relationship between pulmonary function and elevated glycated hemoglobin levels in health checkups: A cross-sectional observational study in Japanese participants

**DOI:** 10.1016/j.je.2016.10.008

**Published:** 2017-01-27

**Authors:** Saigo Baba, Toru Takashima, Miki Hirota, Michihiro Kawashima, Etsuo Horikawa

**Affiliations:** a International University of Health and Welfare, Department of Nursing, Fukuoka, Japan; b Japan Community Health Care Organization (JCHO), Saga Central Hospital Health Administration Center, Saga, Japan; c Japan Community Health Care Organization (JCHO), Saga Central Hospital, Saga, Japan; d Mobility Research Laboratory, Center for Comprehensive and Community Medicine, Graduate School of Medicine, Saga University, Saga, Japan

**Keywords:** Health checkups, Pulmonary function tests, FEV_1_/FVC, Glycated hemoglobin, Diabetes mellitus

## Abstract

**Background:**

Insulin resistance has been associated with cytokines, including interleukin-6 and tumor necrosis factor alpha soluble receptor, both of which are elevated in chronic obstructive pulmonary disease (COPD). Few studies have investigated the relationship between pulmonary function tests using spirometry (PFT) and fasting plasma glucose (FPG) or glycated hemoglobin (HbA_1c_) levels in Japanese participants. The purpose of this study was to clarify the relationship between PFT in Japanese people who had health checkups and their FPG or HbA_1c_ levels. In the context of preventative medicine, we intend to connect early detection of COPD to an index of blood sugar.

**Methods:**

From August 2013 through March 2014, 1019 participants underwent health checkups. PFT, FPG, and HbA_1c_ measurements were conducted. HbA_1c_ levels were measured according to National Glycohemoglobin Standardization Program guidelines.

**Results:**

Participants with FPG ≥100 mg/dL and HbA_1c_ ≥5.6% showed a significantly lower forced expiratory volume in 1 s:forced vital capacity ratio (FEV_1_/FVC) compared to participants with lower FPG and Hb1Ac levels. Prevalence of FEV_1_/FVC values <70% in PFT differed significantly depending on sex, age, body mass index, FPG, HbA_1c_, and smoking habits. Age (≥60 years), HbA_1c_ (≥5.6%), and current or former smoking were associated with FEV_1_/FVC values <70%.

**Conclusion:**

In Japan, HbA_1c_ levels were higher in participants with FEV_1_/FVC values <70% in PFT than in those with FEV_1_/FVC ≥70%. In preventive medicine, PFT by spirometry should be performed in elderly participants with elevated HbA_1c_ levels who are current or former smokers.

## Highlights

• Participants with FEV_1_/FVC values <70% had significantly elevated HbA_1c_ levels.
• Age, HbA1c, and current or former smoking were associated with FEV_1_/FVC values <70%.
• Elderly ever smokers with elevated HbA_1c_ should undergo screened for COPD.

## Introduction

Chronic obstructive pulmonary disease (COPD) is a systemic inflammatory lung disease caused by long-term smoking. Globally, COPD is the fourth-leading cause of death, and in Japan, there is a tendency of high under-diagnosis of patients with COPD.^1^, ^2^ COPD progression leads to inflammation, which is potentially lethal. The main symptoms of COPD are dyspnea and breathlessness during activities. Progression of COPD also has a significant impact on the patient quality of life (QOL). Therefore, early detection and implementation of a suitable treatment for COPD is paramount.

On the other hand, COPD is a systemic inflammatory disease that has been associated with various comorbidities, such as diabetes mellitus (DM), hyperlipidemia (HL), hypertension (HT), and cardiovascular disease.^3^, ^4^, ^5^ DM is a lifestyle-related disease associated with poor diet, such as consumption of high-fat meals, or a lack of exercise, which often manifests as a metabolic syndrome; COPD has been closely linked to smoking history and is considered a major risk factor for DM.^6^, ^7^

Interestingly, smoking has also been related to the development of insulin resistance.^8^, ^9^, ^10^ Additionally, cytokines, such as interleukin-6 and tumor necrosis factor alpha soluble receptor, which are elevated in COPD, have been associated with the development of insulin resistance in liver and muscle tissues; as a result, COPD has been postulated as a risk factor for DM.^11^, ^12^, ^13^, ^14^

A previous study involving 47 million people in a National Hospital Survey investigated the prevalence of comorbidities in patients with COPD and found that the prevalence of DM in patients with COPD was significantly higher than that in patients without COPD.^15^ In addition, a recent large-scale cohort study suggested that COPD was associated with an increased risk of DM, myocardial infarction, and heart failure.^11^, ^16^ Therefore, it is necessary to consider DM management in the treatment of patients with COPD because DM is an important prognosticator in patients with COPD.^17^ A recent meta-analysis indicated that pulmonary function is compromised in patients with DM and pre-diabetes compared to participants with normal fasting plasma glucose (FPG) levels.^18^, ^19^ However, the association between COPD and DM has not yet been elucidated, and few studies have investigated the relationships of pulmonary function tests in people with undiagnosed COPD with FPG or glycated hemoglobin (HbA_1c_) levels.

In Japan, to detect lifestyle-related diseases at an early stage, regular health checkups are recommended for adults aged ≥40 years. The purpose of this study was to clarify the relationships of pulmonary function tests using spirometry (PFT) in people with undiagnosed COPD who had health checkups in Japan with their FPG or HbA_1c_ from the perspective of preventive medicine.

## Methods

In this cross-sectional observational study, participants were selected at the Japan Community Health care Organization (JCHO) Saga Health Administration Center, in Saga Prefecture, Japan. This center provides care for the prevention and early detection of lifestyle-related diseases. Eligible participants included adults aged ≥40 years who attended the center for health checkups from August 2013 through March 2014. Of the 1091 participants enrolled, 72 were excluded because they had physician-diagnosed asthma (n = 52), bronchiectasis (n = 16), or displayed a restrictive abnormality on spirometry (n = 4). Ultimately, we studied 1019 participants. Written informed consent was obtained from all participants. The study was approved by the Institutional Review Board of JCHO Saga Central Hospital and was conducted according to the principles set out in the Helsinki Declaration.

### Pulmonary function tests

Pulmonary function tests, including forced vital capacity (FVC), forced expiratory volume in 1 s (FEV_1_), percent predicted FEV_1_ (%FEV_1_), and the FEV_1_:FVC ratio were measured using conventional spirometry (Chestgraph HI-701; Chest Co., Tokyo, Japan), as recommended by the American Thoracic Society.^20^ According to the Global Initiative for Chronic Obstructive Lung Disease (GOLD),^21^ the FEV_1_/FVC is 70–80% in normal adults, and a value <70% indicates airflow limitation and the possibility of COPD. In adults with an FEV_1_/FVC <70%, GOLD stage I reflects a mild airflow limitation (%FEV_1_ [predicted] ≥80%), stage II is moderate (FEV_1_ ≤50%; %FEV_1_ <80%), stage III is severe (FEV_1_ ≤30%; %FEV_1_ <50%), stage IV is very severe (%FEV_1_ <30% or <50% in conjunction with chronic respiratory failure).^21^ We adopted this GOLD classification in the present study.

### Data for health checkups

Blood samples were obtained for biochemical assays, including FPG and HbA_1c_ assays, which were measured using methods suggested by the National Glycohemoglobin Standardization Program guidelines. FPG levels ≥100 mg/dL or HbA_1c_ levels ≥5.6% indicate pre-diabetes and an increased risk of developing type 2 DM.^22^, ^23^ In this study, we defined participants as having a “high glucose level” if they met either of these criteria. All participants filled out a standard questionnaire on smoking history (Never, non-smoker; Current, current smoker; or Former, past smoker), patterns of physical activity, and a detailed medical history, including any regular medications. Smoking was defined as a dichotomous variable with the answer ‘yes’ to the question ‘Have you smoked at least 100 cigarettes in your entire life?’ This classification combines former and current smokers (with non-smokers as the reference group), and is in line with the definition of smoking status adopted by the United States Centers for Disease Control and Prevention (CDC).^24^

### Analysis

Relationships among sex, age, body mass index (BMI), FPG levels, HbA_1c_ levels, and the presence or absence of DM, HL, HT, and FEV_1_/FVC <70% or FEV_1_/FVC ≥70% were examined using the chi-square (χ^2^) test. Logistic regression analysis was then conducted to identify risk predictors that resulted in an FEV_1_/FVC <70%. The final multivariate model included variables that were retained in a backward analysis, with p < 0.05 indicating statistical significance. All statistical analyses were performed using SPSS version 22.0 (SPSS Japan Inc., Tokyo, Japan).

## Results

A total of 1019 participants' demographics are presented in Table 1. Of the 95 participants with FEV_1_/FVC <70%, 7 (7.4%) had comorbid type 2 DM.

**Table 1:** Participants' demographics (n = 1019).

Characteristics	n (%)	Mean (SD)	(Range)
Sex, male	695 (68.2)		
Age, years		51.7 (8.1)	(40–87)
Body mass index, kg/m^2^		23.2 (3.3)	(13.4–44.0)
**Pulmonary function**			
FVC, L		3.6 (0.8)	(0.5–6.2)
FEV_1,_ L		2.8 (0.6)	(0.8–4.8)
FEV_1_/FVC, %		77.2 (6.1)	(44.1–94.8)
%FEV_1_		89.9 (12.8)	(27.4–138.9)
FEV_1_/FVC <70%	95 (9.3)		
**GOLD stage**			
Stage I	39 (41.1)		
Stage II	56 (58.9)		
**Glycemic control data**			
FPG (mg/dL)		94.5 (17.4)	(62–275)
HbA_1c_ (%)		5.7 (0.7)	(4.4–13.0)
**Smoking status**			
Never	426 (41.8)		
Former	324 (31.8)		
Current	269 (26.4)		
**Disease**			
DM	54 (5.4)		
HL	101 (9.9)		
HT	144 (14.1)		
CVD	16 (1.6)		

CVD, cardiovascular disease; DM, diabetes mellitus; FVC, forced vital capacity; FEV_1_, forced expiratory volume in 1 s; FEV_1_/FVC, ratio of FEV_1_ to FVC; %FEV_1_, percentage of predicted FEV_1_; GOLD, Global initiative for chronic Obstructive Lung Disease; FPG, fasting plasma glucose; HbA_1c_, glycated hemoglobin; HL, hyperlipidemia; HT, hypertension; SD, standard deviation.

**Fig. 1:**
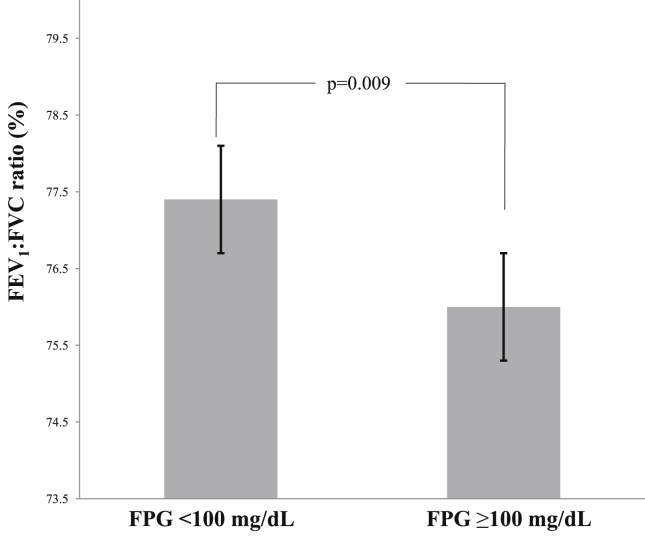
Relationship between fasting plasma glucose level and pulmonary function. The bar graph represents the mean value and the horizontal axis represents the standard deviations of forced expiratory volume in 1 s:forced vital capacity ratios (FEV_1_/FVC). Participants with fasting plasma glucose (FPG) levels of ≥100 mg/dL had significantly lower FEV_1_/FVC compared to those with FPG levels of <100 mg/dL (p = 0.009, Welch T-test).

**Fig. 2:**
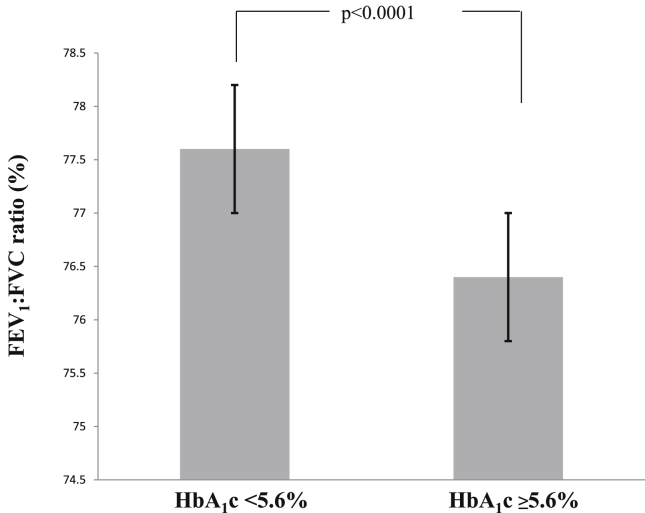
Relationship between glycated hemoglobin level and pulmonary function. The bar graph represents the mean value and the horizontal axis represents the standard deviations of forced expiratory volume in 1 s:forced vital capacity ratios (FEV_1_/FVC). Participants with glycated hemoglobin levels (HbA_1c_) of ≥5.6% had significantly lower FEV_1_/FVC compared to those with HbA_1c_ levels of <5.6% (p < 0.0001, Welch T-test).

Participants with high glucose levels (FPG ≥100 mg/dL) had a significantly reduced FEV_1_:FVC ratio (p = 0.009) (Fig. 1). In addition, participants with high HbA_1c_ levels (≥5.6%) had significantly reduced FEV_1_/FVC (p < 0.0001) (Fig. 2).

Prevalence of FEV_1_/FVC <70% differed significantly according to sex, age, BMI, FPG, HbA_1c_, and smoking status, whereas there was no relationship between the prevalence of FEV_1_/FVC <70% and DM, HL, or HT (Table 2).

**Table 2:** Analysis of risk factors associated with FEV_1_/FVC <70% in all participants (n = 1019).

Factor	FEV_1_/FVC <70%^a^	FEV_1_/FVC ≥70%	*P* value^b^
	(n = 95)	(n = 924)	
**Sex, n**			
Male/Female	74/21	621/303	0.033
**Age, n**			
<60 years/≥60 years	59/36	815/109	<0.001
**Body mass index, n**			
<25 kg/m^2^/≥25 kg/m^2^	79/16	683/241	0.048
**FPG, n**			
<100 mg/dL/≥100 mg/dL	66/29	746/176	0.008
**HbA**_**1c**_**, n**			
<5.6%/≥5.6%	34/61	554/370	0.004
**Smoking status, n**			
Never/Current	25/35	401/234	0.001
Never/Former	25/35	401/289	0.014
Former/Current	35/35	289/234	0.407
**Disease**			
*DM*			
Yes/No	7/88	47/877	0.344
*HL*			
Yes/No	12/83	89/835	0.221
*HT*			
Yes/No	17/78	127/797	0.170
*CVD*			
Yes/No	0/95	16/908	0.206

CVD, cardiovascular disease; DM, diabetes mellitus; FVC, forced vital capacity; FEV_1_, forced expiratory volume in 1 s; FPG, fasting plasma glucose; HbA_1c_, glycated hemoglobin; HL, hyperlipidemia; HT, hypertension.

a FEV_1_/FVC, ratio of FEV_1_:FVC.

b p < 0.05.

Multivariable logistic regression was performed to estimate the risk of FEV_1_/FVC <70% associated with potential predictors, including clinical variables, such as sex, age, BMI, FPG, HbA1c, and smoking status. As a result, logistic regression analysis revealed that age (≥60 years), HbA_1c_ levels (≥5.6%), current smoking, and former smoking were significantly associated with a FEV_1_/FVC <70% (Table 3).

**Table 3:** Logistic regression analysis assessing risk factors associated with FEV_1_/FVC <70%.^a^

Factors	Odds ratio	95% CI^b^	*P* value
**Age, years**			
<60	Reference		
≥60	3.20	1.82–5.63	0.000
**Body mass index, kg/m**^**2**^			
<25	Reference		
≥25	0.27	0.13–0.59	0.001
**HbA**_**1c**_**, %**			
<5.6%	Reference		
≥5.6%	2.04	1.23–3.40	0.006
**Smoking status**			
Never	Reference (or 1.00)		
Former	1.98	1.07–3.67	0.031
Current	3.24	1.71–6.15	<0.001

CI, confidence interval; FVC, forced vital capacity; FEV_1_, forced expiratory volume in 1 s; HbA_1c_, glycated hemoglobin.

a FEV_1_/FVC, ratio of FEV_1_:FVC.

b Logistic regression was used for analysis; Hosmer–Lemeshow test, p = 0.498.

## Discussion

In the present study, participants with high glucose levels (FPG ≥100 mg/dL or HbA_1c_ ≥5.6%) had significantly lower FEV_1_:FVC ratios compared to those who did not. In addition, sex, age, BMI, FPG levels, HbA_1c_ levels, and smoking history were all associated with FEV_1_/FVC <70%. In particular, people with FEV_1_/FVC values <70% had significantly elevated HbA_1c_ levels.

Generally, an FEV_1_:FVC ratio less than 70% on spirometry will result in a diagnosis of COPD. Of the 95 participants with an FEV_1_/FVC <70%, 61 participants showed a HbA1c of more than 5.6% and all had GOLD stage I-II defects (mild-moderate). Only 7.4% of patients with COPD had comorbid DM, which was lower than the 16.3% reported by Schnell et al.^25^ However, the findings of Schnell et al were determined using data from the large-scale NHANES study, which was conducted in the United States and comprised a population of 10 million COPD sufferers. The discrepancy between our study and theirs could be because of significant population differences between Japan and the United States.

A previous meta-analysis suggested an association between COPD and DM,^18^ and it has been reported that pulmonary function is significantly lower in patients with COPD who have comorbidities, such as DM, HL, and HT, than in COPD patients who do not have comorbidities.^26^, ^27^ Despite this, the present study did not show any significant associations of the prevalence of COPD with these comorbidities. This was surprising because previous studies have indicated that the prevalence of DM and COPD comorbidity in patients with high glucose (as indicated by elevated FPG and HbA_1c_) was >10% and that these patients had significantly reduced pulmonary function.^28^, ^29^, ^30^

Based on these results, it was thought that participants who were elderly (≥60 years), had high HbA_1c_ levels, and were current smokers had high tendency to toward impaired pulmonary function. Furthermore, in the present study, the risk of having a FEV_1_/FVC <70% in participants with high HbA_1c_ levels was approximately double that in those with lower HbA_1c_ levels. The present study revealed that FEV_1_/FVC <70% was associated with HbA1c but not for FPG. FPG was determined from blood samples; however, it only indicates the plasma glucose level at that point in time. In contrast, HbA1c provides data reflecting the mean plasma glucose control level over the past 1–3 months. Therefore, it is thought that HbA1c is a better marker as an index of glycemic control. Therefore, from the perspective of preventive medicine, PFT should be conducted when participants undergoing health checkups have elevated FPG or HbA_1c_ levels.

In this study, we stratified the data according to sex in the statistical analysis; however, we were not able to find a sex-related association. It is known that COPD is more common in men; however, in the older populations, the proportion of women is greater because of the high smoking rate among women in Japan.^31^ If we investigate the number of cigarettes smoked (number of packs per year) according to sex in this study, it is likely that there may have been an association between the two. Future studies should investigate the number of cigarettes smoked to determine this.

Our study has several limitations. First, only a small proportion of patients had DM in our study sample. Findings from the 2007 National Health and Nutrition Survey in Japan showed that 15.3% of people were “strongly suspected of having diabetes” as defined using a HbA_1c_ level ≥6.1% or self-reporting of pharmacotherapeutic intake for diabetes.^32^ In our study, the percentage of diabetics with HbA_1c_ levels ≥6.1% was 6.7%, which is low. Many participants showed good glycemic control. Furthermore, a larger sample should be enrolled, and prospective follow-up of participants is necessary. Second, our participants had lower FVC and FEV_1_ values than the general population in Japan.^33^, ^34^ This explains the slightly lower FEV_1_:FVC ratios compared to previous studies. As this study was conducted at a fixed point in time, pre-COPD participants might also be included. Although our result reflects the general tendency of the study participants, there is a limit to grasping the general trend of FEV_1_/FVC values of <70%. Further mechanistic studies are needed to evaluate the relationship between FEV_1_/FVC <70% and glycemic control.

In conclusion, participants with FEV_1_/FVC <70% had significantly elevated HbA_1c_ levels compared to those with FEV_1_/FVC ≥70%. This suggests that, in Japan, people with undiagnosed COPD who have elevated HbA_1c_ levels should undergo spirometry to evaluate pulmonary function for early detection of COPD. In particular, participants aged ≥60 years with elevated HbA_1c_ who are smokers or former smokers should undergo screening for COPD. Early detection of COPD may lead to improved management and reduce medical expenditures.

## Conflicts of interest

None declared.
